# A pilot trial of neoadjuvant pyrotinib plus trastuzumab, dalpiciclib, and letrozole for triple‐positive breast cancer

**DOI:** 10.1002/mco2.505

**Published:** 2024-03-10

**Authors:** Shiwen Huo, Jinqi Xue, Shuo Wang, Huilian Shan, Guanglei Chen, Nan Niu, Yimin Wang, Fang Qiu, Yi Zhao, Fei Xing, Xinyu Zheng, Wei Tu, Ke Li, Hai Zhao, Meiyue Tang, Qianshi Xu, Chao Liu, Yafei Zhao, Xiaofan Jiang, Zheng Pang, Keliang Zhang, Dianlong Zhang, Zhe‐Sheng Chen, Caigang Liu

**Affiliations:** ^1^ Jiangsu Hengrui Pharmaceuticals Co., Ltd. Shanghai China; ^2^ Department of Oncology Shengjing Hospital of China Medical University Shenyang China; ^3^ Innovative Cancer Drug Research and Development Engineering Centre of Liaoning Province Shenyang China; ^4^ Northeastern Clinical Research Alliance of Oncology (NCRAO) Shenyang China; ^5^ Department of Breast Surgery the First Affiliated Hospital of China Medical University Shenyang China; ^6^ Department of Breast Surgery the Forth Affiliated Hospital of China Medical University Shenyang China; ^7^ Department of Breast Surgery Anshan Cancer Hospital Anshan China; ^8^ Department of Breast Surgery Fushun Cancer Hospital Fushun China; ^9^ Center for Drug Evaluation of Liaoning Province Shenyang China; ^10^ Department of Breast Surgery Affiliated Zhongshan Hospital of Dalian University Dalian China; ^11^ Department of Pharmaceutical Sciences College of Pharmacy and Health Sciences St. John's University New York City New York USA

**Keywords:** dalpiciclib, letrozole, neoadjuvant therapy, pyrotinib, trastuzumab, triple‐positive breast cancer

## Abstract

Triple‐positive breast cancer (TPBC) poorly responds to current standard neoadjuvant therapy (trastuzumab plus pertuzumab and chemotherapy). Our previous MUKDEN 01 study showed a promising total pathological complete response (tpCR) rate of 30.4% with neoadjuvant pyrotinib (pan‐human epidermal growth factor receptor tyrosine kinase inhibitor) plus dalpiciclib (cyclin‐dependent kinase 4/6 inhibitor) and letrozole, but the efficacy remains suboptimal. This pilot study (NCT05228951) explored adding trastuzumab to this triplet neoadjuvant regimen in patients with stage II–III TPBC. The primary endpoint was tpCR (ypT0/is, ypN0) rate. Between February 2022 and June 2022, 12 patients were enrolled, and seven (58%; 95% confidence interval [CI], 27.7%–84.8%) patients achieved tpCR. The rate of residual cancer burden (RCB) 0–I was 75% (95% CI, 46.8%–91.1%). The objective response rate (ORR) was 92% (95% CI, 64.6%–98.5%). Mean Ki‐67 level was significantly reduced from 45.0% (95% CI, 19.5%–70.5%) at baseline to 17.2% (95% CI, 0.7%–33.7%) after neoadjuvant therapy (*p* = 0.03). The most common grade 3 adverse events were diarrhea (four [33%]) and decreased neutrophil count (three [25%]). No grade 4 adverse events or treatment‐related deaths occurred. This four‐drug neoadjuvant regimen shows promising pathological response with an acceptable safety profile in patients with TPBC. A randomized controlled trial (NCT05638594) of this regimen is being conducted.

## Graphical abstract

Triple‐positive breast cancer (TPBC) poorly responds to current standard neoadjuvant therapy (human epidermal growth factor receptor 2‐targeted drugs and chemotherapy). This study enrolled 12 TPBC patients to receive pyrotinib, trastuzumab, letrozole, and dalpiciclib. Seven patients achieved total pathological complete response, and the safety profile is tolerable. The preliminary findings are vital for optimizing treatment of TPBC, and prompt further validation in a larger randomized control trial.

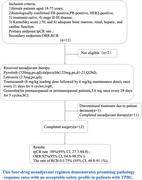

## 1 INTRODUCTION

Human epidermal growth factor receptor 2 (HER2)‐positive breast cancer is an aggressive subtype accounting for 15%–20% of breast cancers.^1^ Emergence of HER2‐targeted agents has revolutionary significance in the treatment of HER2‐positive breast cancer.^2^, ^3^, ^4^, ^5^, ^6^, ^7^, ^8^, ^9^, ^10^, ^11^, ^12^, ^13^, ^14^ The current standard‐of‐care neoadjuvant therapy comprises trastuzumab in combination with pertuzumab plus chemotherapy, which has achieved favorable pathological complete response (pCR) rate.^15^ Data analysis from subgroups suggests that patients with hormone receptor (HR)‐positive, HER2‐positive tumors are less likely to achieve pCR than those with HR‐negative, HER2‐positive tumors, indicating that the interference of estrogen receptor (ER) and/or progesterone receptor (PR) signaling dampens effective control of HER2‐positive breast cancer.^16^

Ample evidence has suggested that HR‐positive, HER2‐positive breast cancer is inherently distinctive from HR‐negative, HER2‐positive breast cancer genetically, resulting in different patterns of treatment response.^17^ Insight into the heterogeneity at genomic level has revealed that the intrinsic subtypes of HER2‐positive breast cancer are distributed to luminal A, luminal B, HER2‐enriched, and basal‐like based on Prediction Analysis of Microarray 50, with around 70% or 80% belonging to luminal subtypes, characterized by the enrichment of luminal gene clusters.^18^ Thus, figuring out the optimal strategy based on comprehension of the complex intrinsic biology characteristics is crucial for treating triple‐positive breast cancer (TPBC). Taken together, the innately different responses to either chemotherapy or targeted therapy between the HR‐positive, HER2‐positive, and HR‐negative, HER2‐positive breast cancer calls for more precise and complete breast cancer proliferation blockade.

As an ideal research hotspot, an increasing number of neoadjuvant trials are under investigation to explore the combination of HER2‐targeted therapy and endocrine therapy in patients with HR‐positive, HER2‐positive breast cancer. Some clinical trials reported modest efficacy with this combination.^19^, ^20^ In the TBCRC 006 study, patients with ER‐positive, HER2‐positive stage II–III breast cancer who received neoadjuvant lapatinib and trastuzumab plus letrozole achieved a total pCR (tpCR) rate of 18% with minimal side effects.^19^ Considering that HER2 and ER function as upstream “mitogen sensor,” dual blockade of them with neglect of downstream mitosis promoting factors may impair treatment efficacy.

Convergence of HER2 and ER pathway on retinoblastoma protein 1 (RB1) forcefully support the necessity of adding cyclin‐dependent kinase (CDK) 4/6 inhibitor to enhance the blockade effect. CDK4/6 has now gradually become a promising target for HER2‐positive breast cancer, as the cyclin D1/CDK4/6/pRb axis is also a key pathway involved in resistance to HER2‐directed therapy.^21^ In the NA‐PHER2 study of patients with ER‐positive, HER2‐positive early or locally advanced breast cancer, neoadjuvant palbociclib and fulvestrant plus trastuzumab and pertuzumab significantly reduced the expression of Ki‐67 and the tpCR rate was 27%.^22^ In our previous MUKDEN 01 study, neoadjuvant pyrotinib (a pan‐HER tyrosine kinase inhibitor [TKI] targeting HER1, HER2, and HER4 that has been approved for the treatment of HER2‐positive early or advanced breast cancer in China)^8^, ^12^, ^14^ plus dalpiciclib (a CDK4/6 inhibitor approved for the treatment of HR‐positive, HER2‐negative advanced breast cancer in China)^23^, ^24^ and letrozole showed a tpCR rate of 30.4% and a residual cancer burden (RCB) −0 or −I rate of 55.7% in patients with stage II–III TPBC.^25^ Promising value of HER2‐targeted therapy and endocrine therapy combined with CDK4/6 inhibitor has been revealed. However, the efficacy in TPBC remains suboptimal.

Here, we conducted this MUKDEN 01 Plus trial to explore the efficacy and safety of trastuzumab combined with the regimen used in MEKDEN 01 (pyrotinib plus dalpiciclib and letrozole)^25^ as neoadjuvant therapy in patients with TPBC.

## 2 RESULTS

### 2.1 Patient characteristics

**FIGURE 1:**
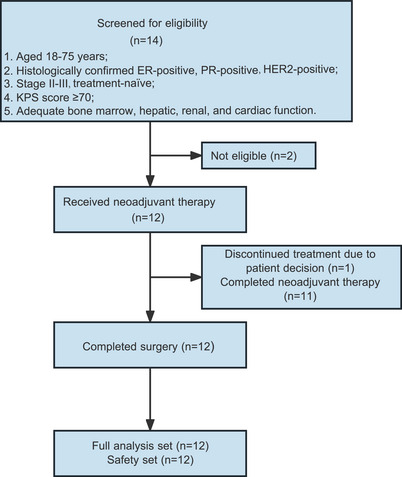
Patient flowchart. ER, estrogen receptor; PR, progesterone receptor; HER2, human epidermal growth factor receptor 2; KPS, Karnofsky performance status.

Between February 16, 2022 and June 2, 2022, a total of 14 patients were screened, and 12 eligible patients were enrolled. Of 12 patients, one discontinued the neoadjuvant therapy after four cycles due to patient decision and underwent surgery; 11 patients completed neoadjuvant therapy and surgery. All these 12 patients were included in the full analysis set and safety set (Figure 1). Baseline characteristics are shown in Table 1. The median age was 52.8 years (range, 41–61). Ten (83%) patients had stage II disease and two (17%) had stage III disease. Eight (67%) patients had lymph node metastases. The mean ER and PR expression levels were 78 ± 16 and 53 ± 36, respectively. The mean Ki‐67 level was 48 ± 22 at baseline.

**TABLE 1:** Baseline patient and disease characteristics.

Characteristics	Patients (*n* = 12)
**Median age, years (range)**	52.8 (41–61)
<55, *n* (%)	7 (58%)
≥55, *n* (%)	5 (42%)
**Menopausal status,** *n* **(%)**	
Premenopausal or perimenopausal	4 (33%)
Postmenopausal	8 (67%)
**Stage at baseline,** *n* **(%)**	
IIA	3 (25%)
IIB	7 (58%)
III	2 (17%)
**Nodal status,** *n* **(%)**	
N0	4 (34%)
N1	6 (50%)
N2	1 (8%)
N3	1 (8%)
**Tumor size,** *n* **(%)**	
T2	11 (92%)
T3–4	1 (8%)
**ER expression (%), mean ± SD**	78 ± 16
**PR expression (%), mean ± SD**	53 ± 36
**Ki‐67 level at baseline (%), mean ± SD**	48 ± 22
**HER2 status,** *n* **(%)**	
IHC 2+/FISH+	6 (50%)
IHC 3+	6 (50%)

Abbreviations: ER, estrogen receptor; FISH, fluorescence in situ hybridization; HER2, human epidermal growth factor receptor 2; IHC, immunohistochemistry; PR, progesterone receptor; SD, standard deviation.

### 2.2 Pathological and radiographic response

**FIGURE 2:**
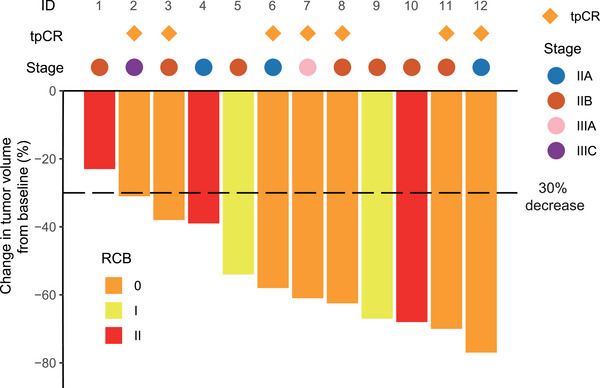
Waterfall plot for radiographic change in tumor volume from baseline (*n* = 12). Pathological response is also shown in this figure. Diamond mark indicates total pathological complete response (tpCR), and different colors of bars indicate residual cancer burden (RCB).

The waterfall plot for radiographic change in tumor volume from baseline and the pathological response are shown in Figure 2. For the primary endpoint, seven (58%; 95% confidence interval [CI], 27.7%–84.8%) of 12 patients achieved tpCR. Nine (75%; 95% CI, 46.8%–91.1%) patients had RCB‐0 or RCB‐I. Regarding radiographic response before surgery, 11 patients achieved partial response, with an objective response rate (ORR) of 92% (95% CI, 64.6%–98.5%).

### 2.3 Change in Ki‐67 level

**FIGURE 3:**
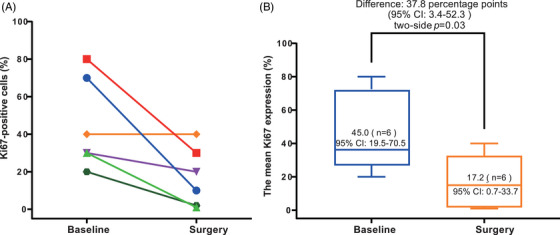
Ki‐67 level at baseline and surgery. (A) Individual data (*n* = 6). (B) Mean percentage of Ki‐67 expressing tumor cells (*n* = 6). The Ki‐67 level in 6 of 12 patients was not assessable due to the lack of detectable tumor cells in the surgical samples. CI, confidence interval.

Due to the lack of detectable tumor cells in the surgical samples, the change in Ki‐67 level was assessed in six patients with available data. Change in Ki‐67 level from baseline to surgery among individual patients and the mean change are shown in Figure 3. The mean Ki‐67 level was 45.0% (95% CI, 19.5%–70.5%) at baseline and then decreased to 17.2% (95% CI, 0.7%–33.7%) after neoadjuvant therapy, with a statistically significant difference (*p* = 0.03).

### 2.4 Safety

Treatment‐emergent adverse events (AEs) are summarized in Table 2. All the 12 enrolled patients (100%) experience at least one any grade AEs. The most common AEs of any grade were decreased neutrophil count (11 [92%]), decreased white blood cell (11 [92%]), diarrhea (11 [92%]), anemia (nine [75%]), oral mucositis (five [42%]), and vomiting (five [42%]). Seven (58%) patients experienced grade 3 AEs, including diarrhea (four [33%]), decreased neutrophil count (three [25%]), decreased white blood cell (one [8%]), and oral mucositis (one [8%]; Table 2). No grade 4 events or treatment‐related deaths occurred. All toxicities were well managed after symptomatic treatment. Dose interruptions of pyrotinib and trastuzumab were required in four (33%) patients, and dose interruption of dalpiciclib was required in two (17%) patients. There were two (17%) patients who required a dose reduction of pyrotinib to 240 mg because of grade 3 diarrhea (*n* = 1) and grade 3 oral mucositis (*n* = 1).

**TABLE 2:** Treatment‐emergent adverse events.

	Patients (*n* = 12)		
Events, *n* (%)	Grade 1 or 2	Grade 3	Grade 4
White blood cell decreased	10 (83%)	1 (8%)	0
Neutrophil count decreased	8 (67%)	3 (25%)	0
Diarrhea	7 (58%)	4 (33%)	0
Anemia	9 (75%)	0	0
Mucositis oral	4 (33%)	1 (8%)	0
Vomiting	5 (42%)	0	0
Alanine aminotransferase increased	3 (25%)	0	0
Fatigue	3 (25%)	0	0
Hypokalemia	3 (25%)	0	0
Aspartate aminotransferase increased	2 (17%)	0	0
Hypocalcemia	2 (17%)	0	0
Nausea	2 (17%)	0	0
Hyponatremia	1 (8%)	0	0
Platelet count decreased	1 (8%)	0	0
Rash	1 (8%)	0	0

### 2.5 Patient‐reported outcomes

**FIGURE 4:**
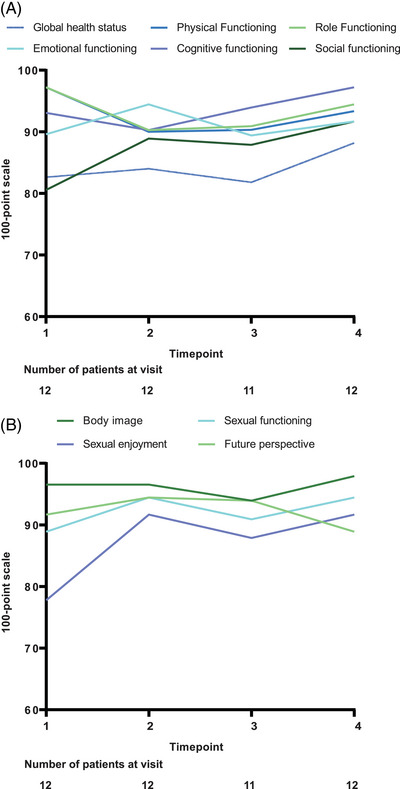
Results of health‐related quality of life questionnaires from baseline to surgery (*n *= 12). (A) Mean scores of global health status and functioning subscales in QLQ‐C30. (B) Mean scores of functioning subscales in QLQ‐BR23. QLQ‐BR23, Quality of Life Questionnaire‐Breast Cancer Module; QLQ‐C30, Quality of Life Questionnaire‐Core 30.

Quality of Life Questionnaire‐Core 30 (QLQ‐C30) and Quality of Life Questionnaire‐Breast Cancer Module (QLQ‐BR23) were used to assess the health‐related quality of life during the treatment period, and all the 12 patients had available results of these two questionnaires. For QLQ‐C30, the role functioning and the physical functioning tended to be reduced after 2 months of neoadjuvant therapy, and was gradually improved to baseline level thereafter. The emotional functioning remained stable throughout the treatment period. The global health status and cognitive functioning tended to be increased along with the treatment. The social functioning showed a clinically meaningful improvement (≥10 points increase) after neoadjuvant therapy (Figure 4A).

For QLQ‐BR23, the sexual enjoyment showed a clinically meaningful improvement after 2 months of neoadjuvant therapy, and remained stable thereafter. The other functioning subscale scores remained stable throughout the treatment period (Figure 4B).

## 3 DISCUSSION

This MUKDEN 01 Plus trial showed that the four‐drug neoadjuvant regimen of trastuzumab plus pyrotinib, dalpiciclib, and letrozole resulted in a tpCR rate of 58% and an ORR of 92% in patients with stage II–III TPBC. Ki‐67 level was significantly reduced after neoadjuvant therapy. The overall safety profile was acceptable. These results indicate that this combination therapy may be a promising alternative neoadjuvant regimen for patients with TPBC.

The outcome of patients with HER2‐positive breast cancer has been dramatically improved since the advent of anti‐HER2 therapy.^2^, ^3^, ^4^, ^5^, ^6^, ^7^, ^8^, ^9^, ^10^, ^11^, ^12^, ^13^, ^14^ Commonly recommended HER2‐targeted drugs include anti‐HER2 monoclonal antibodies (trastuzumab and pertuzumab), HER2 TKIs (neratinib, lapatinib, pyrotinib, and tucatinib), and antibody–drug conjugates (trastuzumab emtansine and trastuzumab deruxtecan).^15^, ^26^, ^27^ Previous clinical trials have established that dual HER2‐targeted blockade with trastuzumab and pertuzumab (or lapatinib) can provide synergistic benefits for patients with HER2‐positive breast cancer.^13^, ^28^, ^29^, ^30^ In the KRISTINE study, the tpCR rate of 18‐week neoadjuvant chemotherapy combined with dual HER2‐targeted agents in patients with HR‐negative, HER2‐positive breast cancer was 73.2%, but that in patients with HR‐positive, HER2‐positive breast cancer was only 43.8%.^13^ The tpCR rate of 18‐week neoadjuvant trastuzumab emtansine plus pertuzumab was also low (35.1%) in the HR‐positive, HER2‐positive subgroup.^13^ In the NeoALTTO study, 12‐week neoadjuvant chemotherapy combined with 18‐week lapatinib and trastuzumab showed a breast pCR rate of 41.6% and 61.3% in the HR‐positive and HR‐negative subgroups,^30^ respectively. In the TRAIN‐2 and BERENICE studies, neoadjuvant chemotherapy combined with trastuzumab and pertuzumab resulted in a tpCR rate of 51.2%–57.3% in patients with HR‐positive, HER2‐positive early or locally advanced breast cancer, which might be attributed to longer neoadjuvant therapy course (20–24 weeks in BERENICE and 27 weeks in TRAIN‐2).^31^, ^32^ However, the significant toxicities associated with long‐course neoadjuvant therapy are major concerns. Novel regimens are needed to be developed.

Previous clinical studies explored HER2‐targeted therapy combined with endocrine therapy in patients with HR‐positive, HER2‐positive breast cancer.^20^, ^33^, ^34^ The PerELISA study evaluated the efficacy of trastuzumab, pertuzumab, and letrozole in molecular responders (Ki‐67 relative reduction >20% from baseline after 2‐week letrozole), and the tpCR rate was 20%.^20^ The TBCRC023 study explored the efficacy of lapatinib plus trastuzumab with letrozole for 12 weeks or 24 weeks in patients with ER‐positive, HER2‐positive breast cancer.^33^ The rate of breast pCR was 9% and 33% following 12‐ and 24‐week therapy,^33^ respectively. The ADAPT study showed that neoadjuvant therapy with 12 weeks of trastuzumab emtansine with or without endocrine therapy, or with 12 weeks of trastuzumab plus endocrine therapy resulted in a tpCR rate of 41.0%, 41.5%, and 15.1% in patients with HR‐positive, HER2‐positive early breast cancer,^34^ respectively. The combination of HER2‐targeted therapy and endocrine therapy does not meet expectation.

Current regimens for HR‐positive, HER2‐positive breast cancer achieve limited efficacy owing to incomplete comprehension on this distinct subtype, calling for persistent treatment optimization. There is a growing interest in CDK4/6 inhibitors for the treatment of HR‐positive, HER2‐positive breast cancer. CDK4/6 inhibitor combined with trastuzumab is effective in transgenic and patient‐derived xenograft mouse models. Moreover, CDK4/6 inhibitors can delay tumor recurrence in a transgenic HER2‐positive breast cancer model.^21^ In the NA‐PHER2 study, the tpCR rate was 27% following neoadjuvant trastuzumab and pertuzumab plus palbociclib, and fulvestrant in patients with ER‐positive, HER2‐positive early or locally advanced breast cancer.^22^ Our previous MUKDEN 01 study investigated the efficacy and safety of pyrotinib plus dalpiciclib and letrozole in patients with stage II–III TPBC.^25^ The results indicated promising efficacy (tpCR rate: 30.4%, rate of RCB 0–I: 55.7%, ORR: 87.4%) of this combination in patients with TPBC.^25^ In this MUKDEN 01 Plus study, we added trastuzumab to the triplet regimen (pyrotinib, dalpiciclib, and letrozole), and this four‐drug regimen resulted in a tpCR rate of 58%, RCB 0–I rate of 75%, and ORR of 92%. It should be noted that our previous MUKDEN 01 study enrolled more patients with stage III disease (36% vs. 17%) and more patients had N2–3 disease (31% vs. 17%) compared with the present MUKDEN 01 Plus study.^25^ Nevertheless, dual HER2‐targeted blockade combined with CDK4/6 inhibitor and endocrine therapy deserves further investigation in TPBC. It has been demonstrated that pan‐HER TKI can enhance the antibody‐dependent cell‐mediated cytotoxicity with additional antiproliferative activity.^35^ Dual HER2‐targeted blockade with small‐molecule pan‐HER TKI plus trastuzumab might be a better option than that with trastuzumab plus pertuzumab. However, our speculations should be interpreted with cautions given the small sample size in our pilot study.

This four‐drug regimen of trastuzumab, pyrotinib, dalpiciclib, and letrozole was relatively safe, and no serious AEs or treatment‐related deaths occurred. Compared with the results from the MUKDEN 01 study,^25^ the addition of trastuzumab led to increased incidence of diarrhea; however, most grade 3 diarrhea events lasted for no more than 2 days and were quickly relieved after management.

We recognized that our study had limitations. This pilot study was conducted in a single center and the sample size was relatively small, leading to inevitable potential bias. In addition, only short‐term efficacy outcomes were reported without long‐term survival results. All these could limit the significance of this study. Our subsequent randomized controlled trial (NCT05638594) is being conducted to validate the efficacy and safety of trastuzumab and pyrotinib plus dalpiciclib and letrozole as neoadjuvant therapy in patients with HR‐positive, HER2‐positive breast cancer, with the head‐to‐head comparator of trastuzumab and pertuzumab plus docetaxel and carboplatin. Both pathological response and survival results will be compared in the future.

In conclusion, neoadjuvant pyrotinib plus trastuzumab, dalpiciclib, and letrozole shows promising pathological response in patients with TPBC, with an acceptable safety profile. This four‐drug neoadjuvant regimen of dual HER2‐targeted blockade with small‐molecule TKI and monoclonal antibody plus CDK4/6 inhibitor and endocrine therapy deserves further validation in randomized controlled trials for patients with TPBC.

## 4 METHODS

### 4.1 Study design and participants

The MUKDEN 01 Plus study was an investigator‐initiated, single‐arm, pilot trial conducted at Shengjing Hospital in China. Patients who met the following inclusion criteria were eligible: (1) female patients aged 18–75 years; (2) histologically confirmed ER‐positive (>10% of tumor cells expressing ER by immunohistochemistry), PR‐positive (>1% of tumor cells expressing PR by immunohistochemistry), HER2‐positive (immunohistochemistry score of 3+, or 2+ with fluorescence in situ hybridization positivity) breast cancer; (3) treatment‐naïve; (4) stage II–III disease according to the 8th edition of American Joint Committee on Cancer Staging Manual^36^; (5) Karnofsky performance status score ≥70; and (6) adequate bone marrow (absolute neutrophil count ≥1.5 × 10^9^/L; platelet count ≥100 × 10^9^/L; hemoglobin level ≥100 g/L), hepatic (total bilirubin ≤1.5 upper limit of normal [ULN]; alanine transaminase and aspartate transaminase ≤3 × ULN), renal (blood urea nitrogen and creatinine ≤1.5 × ULN; creatinine clearance rate ≥50 mL/min [Cockcroft–Gault formula]), and cardiac (left ventricular ejection fraction ≥50%; QT corrected interval ≤480 ms) function. The key exclusion criteria were: (1) bilateral breast cancer, inflammatory breast cancer, or occult breast cancer; (2) other malignant tumors within the past 5 years; (3) serious comorbidities, such as uncontrolled hypertension, diabetes mellitus, and active infection; or (4) pregnant or lactating women.

The study was conducted in accordance with the Declaration of Helsinki and Good Clinical Practice guidelines. The study protocol was reviewed and approved by the ethics committee of Shengjing Hospital (No. 2021PS031T). Written informed consent was obtained from each patient before enrollment. This trial was registered in ClinicalTrials.gov (NCT05228951).

### 4.2 Treatment

Eligible patients received oral pyrotinib (320 mg once daily), oral dalpiciclib (125 mg once daily on days 1–21), and oral letrozole (2.5 mg once daily) every 28 days for five cycles. Intravenous trastuzumab (8 mg/kg loading dose followed by 6 mg/kg maintenance dose) was administrated once every 21 days for six cycles. For premenopausal or perimenopausal patients, goserelin was given by subcutaneous injection at a dose of 3.6 mg once every 28 days for five cycles. Dose interruption, reduction, and discontinuation were permitted for the management of AEs. Secondary prevention with montmorillonite powder (3 g, three times a day) or loperamide (2–16 mg/day) was prespecified for the management of diarrhea. Surgery was performed at 2–4 weeks after the last neoadjuvant therapy. Adjuvant therapy was performed by investigator's discretion according to the National Comprehensive Cancer Network guideline.

### 4.3 Assessments

Tumor samples taken from surgical resection were used for the evaluation of pathological response and RCB according to the Expert Panel Consensus on Pathological Diagnosis of Breast Cancer with Neoadjuvant Therapy, version 2020.^37^ One block per 1 cm of pretreatment tumor size was the sampling principle. If no residual tumor was observed in the sliced specimen, a more extensive sampling was needed. RCB was used as the pathology evaluation criteria, which was calculated based on the area of primary tumor bed, overall cancer cellularity, percentage of cancer that is in situ disease, number of positive lymph nodes, and diameter of the largest metastasis.^38^ RCB was classified as RCB‐0 (no residual disease), RCB‐I (minimal residual disease), RCB‐II (moderate residual disease), and RCB‐III (extensive residual disease).^38^ Imaging examination was performed using magnetic resonance imaging at baseline (within 4 weeks before neoadjuvant therapy), after 2 and 4 months of neoadjuvant therapy, and before surgery. Radiographic response was assessed according to the Response Evaluation Criteria in Solid Tumors, version 1.1. Ki‐67 level was assessed by immunohistochemistry using a Ki‐67‐specific rabbit monoclonal antibody (Ventana, catalog number H36867) at baseline using the biopsy samples and after neoadjuvant therapy using the resected samples.

AEs were monitored from the initiation of neoadjuvant therapy until 28 days after the last neoadjuvant therapy according to the National Cancer Institute Common Terminology Criteria for Adverse Events, version 5.0. Blood routine was done at days 0, 7, and 14 of the first neoadjuvant therapy cycle, day 0 for the subsequent cycles, and before surgery. Blood biochemistry and electrocardiogram were done before each cycle and surgery. Urine and stool routines and coagulation function test were done when necessary. Self‐reported discomfort by patients using log card was also collected.

Health‐related quality of life was assessed using the European Organization for Research and Treatment of Cancer QLQ‐C30^39^ and QLQ‐BR23^40^ at baseline, after two and four cycles of neoadjuvant therapy, and before surgery. The mean score of each subscale in QLQ‐C30 and QLQ‐BR23 was standardized to a range of 0–100 points. Higher scores of global health status in QLQ‐C30 and functioning subscales in QLQ‐C30 and QLQ‐BR23 indicated better status.

### 4.4 Endpoints

The primary endpoint was tpCR (ypT0/is, ypN0) rate, defined as no residual tumor cells in the breast and axillary lymph nodes.

Secondary endpoints were ORR (defined as the proportion of patients with the best response of complete or partial response) before surgery, breast pCR (ypT0/is; defined as no residual tumor cells in the breast) rate, RCB, change in Ki‐67 level from baseline to surgery, and safety. The exploratory endpoint was health‐related quality of life.

### 4.5 Statistical analysis

Efficacy was analyzed in the full analysis set, defined as all patients with at least one dose of the study drug. Safety was analyzed in the safety set, defined as all patients who received at least one dose of the study drug and had available safety data.

Continuous data were expressed as mean ± standard deviation, mean (95% CI), or median (range), while categorical data were expressed as frequency (percentage). The 95% CIs of tpCR rate, breast pCR rate, the rate of RCB‐0 or ‐I, and ORR were estimated using the Clopper–Pearson method. Ki‐67 levels at baseline and after neoadjuvant therapy were compared using the paired *t*‐test. All statistical analyses were performed using SAS 9.4 (SAS Institute). Two‐sided *p* < 0.05 was considered statistically significant.

## AUTHOR CONTRIBUTIONS

Conception and design: Caigang Liu, Zhe‐Sheng Chen, and Shiwen Huo. Acquisition of data: Jinqi Xue, Guanglei Chen, Shuo Wang, Huilian Shan, Nan Niu, and Meiyue Tang. Analysis and interpretation of data: Jinqi Xue, Nan Niu, Yimin Wang, Qianshi Xu, Chao Liu, Yafei Zhao, Xiaofan Jiang, and Keliang Zhang. Drafting of the manuscript: Nan Niu. Critical revision of the manuscript for important intellectual content: Caigang Liu. Administrative, technical, or material support: Jinqi Xue, Guanglei Chen, Shuo Wang, Dianlong Zhang, Fang Qiu, Yi Zhao, Fei Xing, Xinyu Zheng, Wei Tu, Ke Li, Hai Zhao, Zheng Pang, and Shiwen Huo. Study supervision: Caigang Liu. Approval of the final version of manuscript for submission: All authors.

## CONFLICT OF INTEREST STATEMENT

Shiwen Huo and Zheng Pang are employees of Jiangsu Hengrui Pharmaceuticals Co., Ltd. Zhe‐sheng Chen is the member of the editorial board of MedComm, he was not involved in the journal's review or decision related to this manuscript.  Other authors declare no conflicts of interest.

## ETHICS STATEMENT

The study was conducted in accordance with the Declaration of Helsinki and Good Clinical Practice guidelines, and was approved by the ethics committee of Shengjing Hospital (No. 2021PS031T). Written informed consent was obtained from each patient. This trial has been registered in ClinicalTrials.gov (NCT05228951).

## Data Availability

The datasets generated and/or analyzed during the current study are available from the corresponding author Caigang Liu on request for 10 years; deidentified clinical data and experimental data will be shared after the approval of the institutional ethics committee.
